# Fixed-bed column recirculation system for investigation of sorption and biodegradation of organic pollutants in saturated sediment: a detailed design and development

**DOI:** 10.1186/s40064-016-3551-0

**Published:** 2016-10-21

**Authors:** Bao Son Trinh, Brian Reid, Kevin Hiscock

**Affiliations:** 1 Institute for Environment and Resources, Vietnam National University of Hochiminh City, 142 To Hien Thanh Street, District 10, Ho Chi Minh City, Vietnam; 2 School of Environmental Sciences, University of East Anglia, Norwich Research Park, Norwich, NR4 7TJ UK

**Keywords:** Sorption, Biodegradation, Fixed-bed column recirculation, Sediment, Organic pollutant

## Abstract

**Background:**

Sorption and biodegradation are the primary processes of organic pollution remediation in aquatic and soil/sediment environments. While researchers have substantially reported their findings regarding these processes, little attention has been given to description of experimental apparatus. This technical paper aims to present the development and detailed design of a fixed-bed column recirculation (FBCR) system which has been widely applied to investigate sorption and biodegradation of organic pollutants in aquatic and/or sediment environments.

**Findings:**

The FBCR system was developed and tested by three experiments investigating sorption and biodegradation of two herbicides (isoproturon and mecoprop) in different saturated materials (hydrofilt and river sediment). Efficiency of the FBCR system was assessed according to criteria i.e. reliability, leaking inhibition, reproducibility, practical of use and cost. The results indicated that the latest version (Version 4) of the FBCR system has been significantly improved and ready to extend to similar studies.

**Conclusions:**

This system is therefore recommended to researchers who intend to investigate the remediation of organic pollutants in aquatic, soil and sediment environments.

**Electronic supplementary material:**

The online version of this article (doi:10.1186/s40064-016-3551-0) contains supplementary material, which is available to authorized users.

## Background

**Fig. 1:**
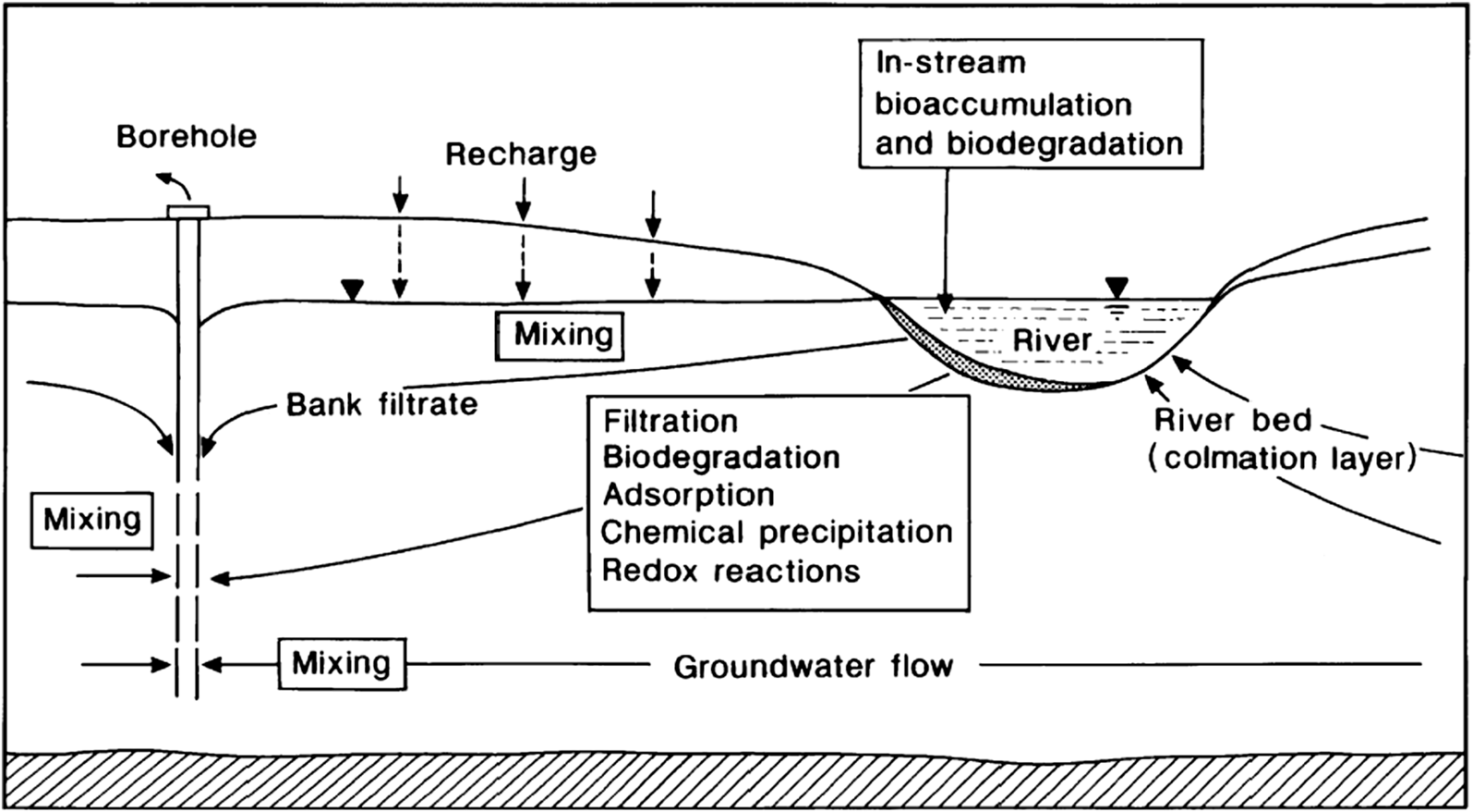
Schematic diagram of processes affecting water quality during bank filtration (Hiscock and Grischek 2002)

In aquatic and riverbed environments, various processes such as sorption, biodegradation, redox reactions can directly and/or indirectly influence on both surface water and groundwater quality (Fig. 1) (Hiscock and Grischek 2002). Sorption and biodegradation processes, however, have been reported as the primary processes which can reduce and totally discompose organic pollutants into carbon dioxide and water (Karickhoff et al. 1979; Karickhoff 1984; Bornick 1998; Cornelissen et al. 2005; Katagi 2006, Trinh et al. 2012). Along with different methods to investigate these processes e.g., shaking batch, immobilised bioreactors, packing column, etc., (Alexander 1999), the fixed-bed column recirculation (FBCR) system, also known as a ‘test-filter’, was firsly introduced by Sontheimer (1988). It was then widely applied to monitor the attenuation of organic pollutants in river water and river sediment (Sontheimer 1988; Knepper et al. 1999; Bornick et al. 2001; Trinh et al. 2012). In these contexts, the columns were packed with river sediment and contaminated water was then recirculated through the system. The main advantages of the FBCR system are (1) the infiltration flow rate can be controlled by peristaltic pump and (2) the desired contact time (in case of limited column length) can be achieved by recirculation period. While many FBCR systems have been successfully applied to investigate sorption and biodegradation of organic pollutants in soil/sediment (Sontheimer 1988; Knepper et al. 1999; Bornick et al. 2001; Trinh et al. 2012), little attention has been given to description of how to technically reproduce and efficiently apply this system. This technical paper therefore aims to present the development and detailed design of a FBCR system which can support for investigation of sorption and biodegradation of organic pollutants in soil/sediment. Three testing experiments with two herbicides (isoproturon and mecoprop) recirculating through the saturated river sediment columns are also presented to demonstrate the efficiency and reliability of this FBCR system.

## Development of a FBCR system

**Fig. 2:**
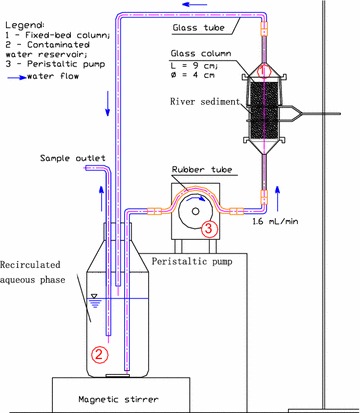
Schematic diagram of a fixed-bed column recirculation system and experimental set-up

**Fig. 3:**
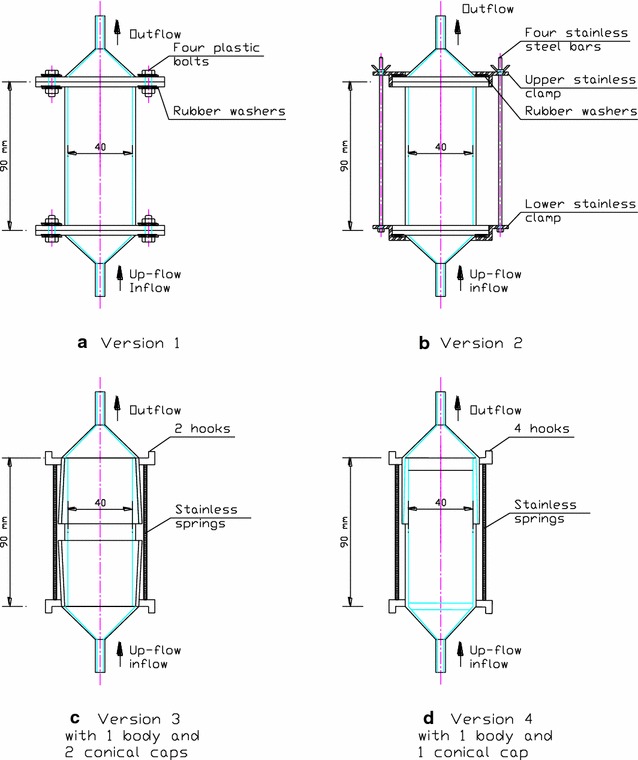
Designed drawings of the four versions of the fixed-bed column recirculation system: (**a**) Version 1: a body connected two end-caps by plastic bolts and nuts; (**b**) Version 2: a body connected two end-caps by stainless steel clamps; (**c**) Version 3: a body connected two conical caps by conical joints and springs; and (**d**) Version 4: a body connected one conical cap by conical joints and springs

A diagram of the FBCR system developed in this study is presented in Fig. 2. The column is the most important unit of the system. It was designed and developed based on the principles and conceptual models of the previous fixed-bed column bioreactors (Sontheimer 1988; Knepper et al. 1999; Bornick et al. 2001). Glass was chosen as the material to make the column because of its inert property. Dimensions of the column were determined to be 40 mm in diameter and 90 mm in length based on simulation of the in situ processes. Figure 3 presents four versions of the columns which were developed for investigation of sorption and biodegradation of the organic pollutants in river sediment.

In Version 1 (Fig. 3a), the body and the two end-caps were connected together by eight sets of plastic bolts and nuts (four at each end). The column was packed with Hydrofilt material (see the Additional file 1). The system worked properly without leakage at low internal pressure (due to large particle sizes of Hydrofilt, from 0.8 to 1.5 mm). However, once river sediment was packed in the column, internal pressure increased considerably due to fine particle size of the sediment (23 % silt and 76 % sand, Additional file 1: Table S2). This resulted in leakage and, in several cases once the plastic bolts were tightened, fracture was observed. Leakage was often found at the connection between the body and the end-cap. In addition, the cap and the body were easily broken once the plastic bolts were tightened in connecting these parts together.

Version 2 (Fig. 3b) was created to improve the shortcomings of Version 1. Two stainless steel clamps were made to house the body and the two end-caps by using four stainless steel studdings and wing nuts holding the clamps. This design improved the leakage problem and minimized incidences of breakage. However, this design was inconvenient for packing sediment material because the lower end-cap and the body were not fixed together once the upper end-cap was taken off for packing. Furthermore, manufacturing of the stainless steel clamps was costly.

In Version 3 (Fig. 3c), a number of improvements were applied. Two conical connections at the two ends of the column were designed to replace the clamp-connections used in Version 2. The two caps and body of the column were connected by the two conical joints (vaseline should be used at the connect contact in order to facilitate the disassembling step). The connection was strengthened by stainless steel springs (or rubber bands) and hooks surrounding four sides of the column. This design also improved the manipulation of packing material in the column, making it quicker and easier to use. The leakage problem was mostly prevented by the conical joint. After several tests, the advantages of this version were compared to the previous versions and noted in terms of its faster manufacture, easier manipulating and packing, and lower production cost. Experiencing the trials, however, it was observed that the lower conical cap was not really necessary and, as a consequence, further refinements were made resulting in the final version (Version 4).

**Fig. 4:**
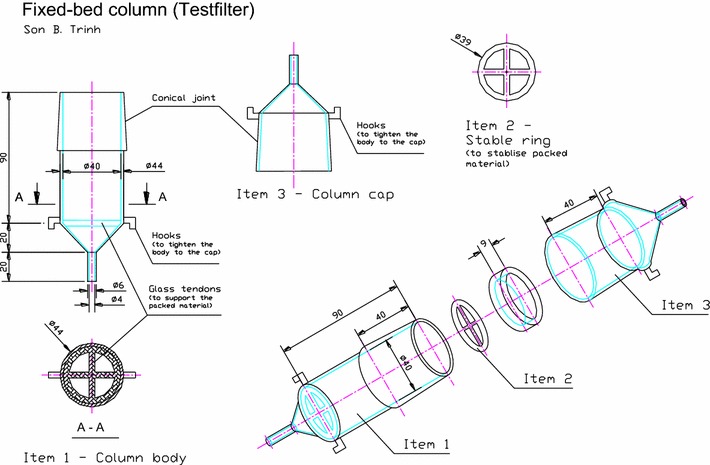
A detailed drawing of the column for the fixed-bed column recirculation system (Version 4). Dimensions are given in mm

In Version 4 (Fig. 3d), the lower conical joint and the column body as presented in Version 3 were replaced by a combined single section of the column. Removable glass tendons were also designed and placed at the two ends of the column in order to support the filter paper and therefore fix the packing material (Item 2, Fig. 4). In this version, packing material step was easier and quicker through the only upper conical cap. The body of the column and the upper conical cap can be assembled by stainless steel springs (or rubber bands) and surrounding hooks. These improvements has facilitated the packing step, reduced the cost (by reducing one conical cap), and further saved time in terms of production. Figure 4 shows the final design of Version 4 of the fixed-bed column.

## Materials and methods for testing the FBCR system

### Materials

River water and river sediment were collected at River Thames (Gatehampton, Reading, UK). Hydrofilt, a porous material used in water treatment, was purchased from Akdolit^®^ Company. Herbicides isoproturon (IPU) (3-(4-isopropylphenyl)-1,1-dimethyl urea) and mecoprop (MCPP) (2-(4-chloro-o-tolyloxy) propaonic acid) were purchased from Sigma Aldrich, UK. Further information of these materials, their characteristics, and analytical procedure are presented in Additional file 1: Tables S1 and S2.

### Methods

Three experiments were established to test the FBCR system.

#### Experiment 1: Testing IPU attenuation in Hydrofilt material with Version 1

**Fig. 5:**
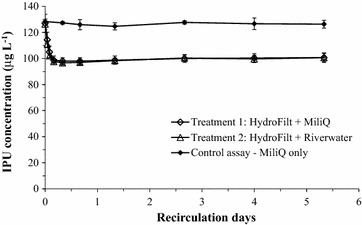
Testing the fixed-bed column recirculation system with Version 1: flux of isoproturon (IPU) in HydroFilt sorbent (Experiment 1). *Error bars* represent standard error of three replicates

Hydrofilt (70 g) was packed in the columns. MiliQ water and river water (1.5 L) were transferred to each reservoirs of Version 1 to establish Treatments 1 and 2 (n = 3), respectively. A controlled treatment (n = 3) was also prepared with the empty column (no packing material) and reservoir filled with MiliQ water (1.5 L). The IPU stock solution was spiked in every reservoirs to achieve the final concentration of about 130 μg L^−1^. The contaminated solutions were recirculated through the columns for 128 h (over 5 days). Aqueous samples were collected from the reservoirs at every intervals (Fig. 5). The samples were then analysed by the HPLC system to determine IPU concentration.

#### Experiment 2: Testing IPU attenuation in river sediment with Version 3

River sediment (150 g) was packed in the columns. River water (1.5 L) was transferred to the reservoir of Version 3. The IPU stock solution was spiked in the reservoir to achieve final concentration of about 1050 μg L^−1^. The contaminated solution was recirculated through the column for 14 days. Aqueous samples were collected from the reservoir at every intervals. The samples were then analysed by the HPLC system to determine IPU concentration.

#### Experiment 3: Testing MCPP attenuation in river sediment with Version 4

River sediment (150 g) was packed in the columns. River water (1.5 L) was transferred to the reservoirs of Version 4 system. Two treatments (n = 3) were established: Treatment 1 with both sterilised river water and river sediment (autoclaved at 121 °C for 30 min); and Treatment 2 with both non-sterile river water and river sediment (the natural materials). The MCPP stock solution was spiked in the reservoirs to achieve the final concentration of about 100 μg L^−1^. The contaminated solution in the reservoir was recirculated through the column for 18 days. Aqueous samples were collected at every intervals. The samples were also analysed by HPLC to determine MCPP concentration.

## Results and discussion

### Sorption of IPU on Hydrofilt material (Experiment 1)

IPU concentration in the controlled treatment (without packing material) did not significantly decrease (p > 0.05) over 128 h of recirculation (Fig. 5). This indicated that IPU was not significantly lost by sorption to the wall of the apparatus or by evaporation. IPU concentrations in both Treatments 1 and 2 were observed as a biphasic model: (1) the *rapid sorption phase* occurred over the initial one-third day; and (2) the *stable phase* occurred over the remaining of 5 days. Over the rapid sorption phase, IPU concentration significantly decreased (15 %) in both Treatments 1 and 2. Stable phase indicated that the equilibrium state of IPU between the solid and liquid phases could be reached. Several isotherm sorption parameters of IPU on Hydrofilt were estimated i.e. the maximum sorption capacity of Hydrofilt to IPU (C_S,max_) to be 614 ± 71 µg kg^−1^ and the solid-water distribution coefficient (*K*_*d*_) to be 6.28 ± 0.70 L kg^−1^.

### Sorption and biodegradation of IPU in river water–river sediment system (Experiment 2)

**Fig. 6:**
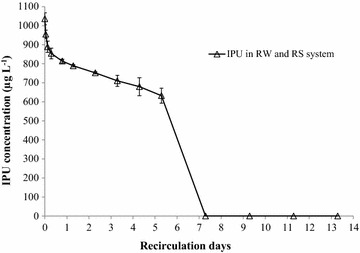
Testing the fixed-bed column recirculation system with Version 3: flux of isoproturon (IPU) in river sediment (Experiment 2). *Error bars* represent standard error of three replicates. *RW* river water, *RS* river sediment

IPU concentration in this treatment was observed as a triphasic model (Fig. 6): (1) the *rapid sorption phase* occurred over the first two-third day; (2) the *stable phase* or *adaptation phase* occurred over the following 5 days; and (3) the *biodegradation phase* occurred over the remaining of 2 days. Over the rapid sorption phase, a decrease of 20 % of IPU in aqueous solution was observed. Assuming sorption equilibrium was reached after the initial two-third day, isotherm sorption parameters of IPU on river sediment were estimated i.e. C_S,max_ = 3174 ± 218 µg kg^−1^; K_d_ = 4.22 ± 0.28 L kg^−1^; organic carbon-normalised partition coefficient, K_OC_ = 527.16 ± 34.91 L kg^−1^; and retardation factor, R_D_ = 11.4 ± 0.7. Over the *adaptation phase*, an addition of 15 % of IPU concentration decreased over 5 recirculation days. This observation could be explained by slow degradation rate of microorganism in river sediment and river water during the adaptation or lag period. After this period, the population of the potential IPU degrading microorganisms could significantly increase. As a consequence, the IPU degradation rate significantly increased. Over the *biodegradation phase* (2 circulation days), it was observed that IPU rapidly decreased (from 650 ± 39 to below 1 µg L^−1^). This result suggests that microorganisms in river sediment could be the main factor to reduce IPU in river water. Similar observations were also reported by previous authors (Walker et al. 2001; Bending et al. 2006; Trinh et al. 2012).

### Sorption and biodegradation of MCPP in river water–river sediment system (Experiment 3)

**Fig. 7:**
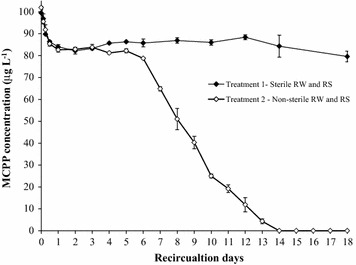
Testing the fixed-bed column recirculation system with Version 4: flux of mecoprop (MCPP) in river sediment (Experiment 3). *Error bars* represent standard error of three replicates. *RW* river water, *RS* river sediment

MCPP concentration in Treatments 1 and 2 of Experiment 3 was presented in Fig. 7. In Treatment 1 (sterile treatment), a biphasic model was observed with (1) the *rapid sorption phase* occurred over the first day and (2) the *stable phase* occurred during the remaining circulation period. Notwithstanding, MCPP concentration in Treatment 2 (the non-sterile treatment) was observed as a triphasic model: (1) the *rapid sorption phase* occurred over the first circulation day; (2) the *stable phase* or *adaptation phase* occurred over the next 4 days; and (3) the *biodegradation phase* occurred over the remaining of 9 circulation days. Similar to the previous calculation for IPU, several sorption parameters of MCPP in river sediment were also estimated i.e. C_S,max_ = 248 ± 27 µg kg^−1^; K_d_ = 2.98 ± 0.35 L kg^−1^; K_OC_ = 373 ± 44; R_D_ = 8.37 ± 0.87. Treatment 2 showed that *adaptation phase* was about in 2 and 5 days with low decrease of MCPP observed during this period. However, MCPP concentration significantly decreased (from 83 ± 1 μg L^−1^ to below 2 μg L^−1^) over the *biodegradation phase* (the following 9 days). Along with the observation in Experiment 2, this result again indicates that microorganisms in river sediment could be the main factor to degrade MCPP. This observation is consistent with the previous findings (Lappin et al. 1985; Albrechtsen et al. 2001; Hoppe-Jones et al. 2010; Trinh et al. 2012).

## Conclusion

Observations from the three experiments indicated that the FBCR system is reliable and useful for investigation of sorption and biodegradation of organic pollutants in saturated sediment. This system was found to be efficient, reproducible, practical in terms of packing materials and leaking control, low cost and quick to assemble. In addition, unnecessary mistakes presented in this paper could be also helpful in terms of saving time and cost to reproduce this system. It is therefore recommended to researchers who intend to investigate sorption and biodegradation processes of organic pollutants in aquatic, soil and sediment environments.

## Additional file

**Additional file 1: Supporting information**. Further details of sampling location and method, physical-chemical properties of the materials, analytical procedures, and sorption calculation method are presented in the Supporting Information. This material is available online and free of charge.
