# Supplementary material for: Fixed-bed column recirculation system for investigation of sorption and biodegradation of organic pollutants in saturated sediment: a detailed design and development
Source: Springerplus. 2016 Oct 21;5(1):1842. doi: 10.1186/s40064-016-3551-0 (PMC5074949; doi:10.1186/s40064-016-3551-0)
Supplement: Supplementary file 1 — Additional file 1: Supporting information. Further details of sampling location and method, physical-chemical properties of the materials, analytical procedures, and sorption calculation method are presented in the Supporting Information. This material is available online and free of charge. [file 40064_2016_3551_MOESM1_ESM.docx]

(**Supporting Information**)

fixed-bed column recirculation method for iNvestiGATION of sorption and biodegradation of organic pollutants in saturated sediment: a detailed design and development

Bao Son Trinh^†,‡*^, Brian Reid^‡^, Kevin Hiscock^‡^,

^†^ Institute for Environment and Resources, Vietnam National University of Hochiminh City, 142 To Hien Thanh street, District 10, Ho Chi Minh city, Vietnam

^‡^ School of Environmental Sciences, University of East Anglia, Norwich Research Park, Norwich NR4 7TJ, UK

* Corresponding Author: trinhbao_son@yahoo.com

*Materials and reagents*

River sediment (RS) was collected from the River Thames (N51^o^ 30.850’; W001^o^ 08.301’, Reading, UK) by crew hand auger. Sediment (1 kg) were taken from nine positions: threes at 1 m distance from the bank (water level 0.3 m); the other threes at 4 m from the bank (water level 0.6 m); and the last threes at 7 m from the bank (water level 0.9 m). These samples were then mixed together to produce a single composite sample. River water (RW) (10 L) was collected at the same position of RS and stored in a polypropylene can. The samples were transported to the University of East Anglia’s laboratory in the same day and stored in the cold room (4 ^o^C). Tables S1 and S2 present the physical-chemical properties of the RS and RW samples.

*Reagents*

HydroFilt, a porous, siliceous filter material for water treatment with grain size 0.8 – 1.5 mm, was purchased from Akdolit® Company. Isoproturon (IPU) and Mecoprop (MCPP) (99.8 %) were of analytical grade purchased from Sigma Aldrich, UK. MCPP and IPU stock solutions (10 mg L^-1^) were prepared in MiliQ water, then sonicated for 30 minutes. Other chemicals were reagent grade and obtained either from Sigma Aldrich or Fisher Scientific (Leicestershire, UK).

*Analytical procedure by high performance liquid chromatographic (HPLC) instrument*

Pesticides IPU and MCPP were measured by the Dionex Summit HPLC (main column: Acclaim 120 C18 5 μm 120 Ǻ Dionex, 250 x 2.1 mm; guard column; detector: PDA-100 photodiode array). Running modes were: mobile phases acetonitrile (A) and NaHPO_4_ (B) mixed with an isocratic mode of 50 % A and 50 % B; flow-rate of 0.4 mL min^-1^; injection volume of 180 μL; column temperature of 25 ^o^C; UV detection for MCPP and IPU recorded at wavelengths of 230 nm and 242 nm, respectively. The Chromeleon software (version 6.8, service package 5) was applied to return the pesticide concentration. The limit of detection for analysing MCPP and IPU by this HPLC procedure was determined to be of 2 and 1 µg L^-1^, respectively.

*Statistical methods*

Analysis of variance (ANOVA) and/or paired sample t-tests were used to compare differences between treatments. For all tests, a significance p-value of less than 0.05 was used. All statistical analyses were undertaken using Microsoft Office Excel 2007, SPSS for Windows^®^ (version 16.0) and Sigma Plot 2000.

**Table S1:** Physico-chemical properties of river water (RW) samples: RW1 collected on 14 September, 2007 and RW2 collected 18 April, 2008. Errors where shown are standard errors (n = 3).

| **Parameters** | **RW1** | **RW2** |
| --- | --- | --- |
| Temp. (^o^C) | 16.8 | 10.6 |
| pH | 8.12 ± 0.01 | 8.35 ± 0.01 |
| Elect. cond. (µS cm^-1^) | 867 ± 1 | 770 ± 24 |
| Diss. oxygen (mg L^-1^) | 9.2 ± 0.5 | 9.1 ± 1.1 |
| Total nitrogen (mg L^-1^) | 8.4 ± 0.1 | 9.2 ± 0.4 |
| Total carbon (mg L^-1^) | 51.3 ± 0.4 | 31.7 ± 0.6 |
| Total organic carbon (mg L^-1^) | 50.4 ± 0.5 | 26.8 ± 0.7 |
| Alkalinity (mEq L^-1^) | 3.38 ± 0.08 | 4.44 ± 0.03 |
| HCO_3_^-^ (mg L^-1^) | 260 ± 5 | 256 ± 23 |
| Cl^-^ (mg L^-1^) | 25.8 ± 2.5 | 30.5 ± 1.4 |
| NO_3_^-^ (mg L^-1^) | 25.0 ± 1.2 | 25.2 ± 0.3 |
| SO_4_^2-^ (mg L^-1^) | 46.4 ± 3.6 | 50.2 ± 3.3 |
| Na^+^ (mg L^-1^) | 27.0 ± 1.0 | 19.1 ± 0.8 |
| Ca^2+^ (mg L^-1^) | 100.5 ± 5.1 | 84.6 ± 4.3 |
| Mg^2+^ (mg L^-1^) | 4.98 ± 0.5 | 3.78 ± 0.5 |
| K^+^ (mg L^-1^) | 5.60 ± 0.6 | 3.11 ± 0.3 |

**Table S2:** Physico-chemical properties of the river sediment (RS) samples: RS1 collected on 14 September, 2007 and RS2 collected on 18 April, 2008. Errors where shown are standard errors (n = 3).

|  | **RS1** | **RS2** |
| --- | --- | --- |
| Particle size fractionation, (%)  50 – 2000 µm (sand)  2 – 50 µm (silt)  0.020 – 2 µm (clay) | 76  23  1 | 79  20  1 |
| pH | 8.29 ± 0.05 | 8.35 ± 0.06 |
| Density (g cm^-3^) | 2.65 ± 0.00 | 2.72 ± 0.08 |
| Bulk density (g cm^-3^) | 1.25 ± 0.02 | 1.31 ± 0.02 |
| Porosity (%) | 50.6 ± 2.1 | 51.3 ± 1.8 |
| Surface area (m^2^ g^-1^) | 0.071 ± 0.003 | 0.079 ± 0.004 |
| Total nitrogen (% w/w) | 0.45 ± 0.01 | 0.05 ± 0.01 |
| Total carbon (mg L^-1^) | 6.21 ± 0.22 | 4.42 ± 0.19 |
| Total organic carbon (mg L^-1^) | 0.94 ± 0.07 | 0.80 ± 0.07 |
| Total sulphur (mg L^-1^) | 0.43 ± 0.03 | 0.10 ± 0.03 |

*Calculation procedure for sorption parameters*

Sorption parameters were calculated based on the initial and equilibrium concentrations of the compounds.

- 1. *Maximum sorption capacity* on RS*, C_S,max_ (μg kg^-1^)*: the maximum amount of sorbate (μg) associated with an amount of dry sorbent (RS) (kg);

$C_{S,\max}=\frac{(C_{o}-C_{e})*V_{W}}{m_{dry,sed}}$ (μg kg^-1^) (1)

where

C_o_ – initial concentration of sorbate in the aqueous phase, μg L^-1^;

C_e_ – equilibrium concentration of sorbate in the aqueous phase, μg L^-1^.

V_W_ – volume of RW, L;

m_dry,sed_ – mass of dry sorbent (RS), kg.

- 1. *Solid-water distribution coefficient, K_d_ (*L kg^-1^*)*: ratio of C_S, max_ and C_e_:

$K_{d}=\frac{C_{S,\max}}{C_{e}}$ (L kg^-1^) (2)

- 1. *Organic carbon-normalised distribution coefficients, K_OC_*: ratio of K_d_ and the mass fraction of organic carbon:

$K_{OC}=\frac{K_{d}}{f_{OC}}$ (3)

where

ƒ_OC_ – fraction of organic carbon by mass in sediment.

- 1. *Retardation factor, R_D_*:

$R_{D}=1+K_{d}\frac{\rho_{b}}{n}$ (4)

where

*ρ_b_* – bulk density of the sorbent (RS), kg L^-1^;

*n* – porosity of the sorbent (RS), %.
